# Integrated Machine Learning and Chemoinformatics-Based Screening of Mycotic Compounds against Kinesin Spindle ProteinEg5 for Lung Cancer Therapy

**DOI:** 10.3390/molecules27051639

**Published:** 2022-03-02

**Authors:** Priyanka Maiti, Priyanka Sharma, Mahesha Nand, Indra D. Bhatt, Muthannan Andavar Ramakrishnan, Shalini Mathpal, Tushar Joshi, Ragini Pant, Shafi Mahmud, Jesus Simal-Gandara, Sultan Alshehri, Mohammed M. Ghoneim, Maha Alruwaily, Ahmed Abdullah Al Awadh, Mohammed Merae Alshahrani, Subhash Chandra

**Affiliations:** 1 Centre for Environmental Assessment and Climate Change, G.B. Pant National Institute of Himalayan Environment (GBP-NIHE), Kosi-Katarmal, Almora 263643, Uttarakhand, India; priyankamaiti.06@gmail.com; 2 Department of Botany, DSB Campus, Kumaun University, Nainital 263002, Uttarakhand, India; priyancisharma@gmail.com; 3 ENVIS Centre on Himalayan Ecology, G.B. Pant National Institute of Himalayan Environment (GBP-NIHE), Kosi-Katarmal, Almora 263643, Uttarakhand, India; 4 Centre for Biodiversity Conservation and Management, G.B. Pant National Institute of Himalayan Environment (GBP-NIHE), Kosi-Katarmal, Almora 263643, Uttarakhand, India; bhatt_id@rediffmail.com; 5 ICAR-Indian Veterinary Research Institute, Bengaluru 560024, Karnataka, India; maramakrishnan@gmail.com; 6 Department of Biotechnology, Bhimtal Campus, Kumaun University, Nainital 263136, Uttarakhand, India; shalinimathpal.121@gmail.com (S.M.); tjoshi6869@gmail.com (T.J.); raginipant1@gmail.com (R.P.); 7 Genetic Engineering and Biotechnology, University of Rajshahi, Rajshahi 6205, Bangladesh; shafimahmudfz@gmail.com; 8 Nutrition and Bromatology Group, Department of Analytical Chemistry and Food Science, Faculty of Science, Universidade de Vigo, E-32004 Ourense, Spain; jsimal@uvigo.es; 9 Department of Pharamaceutics, College of Pharmacy, King Saud University, Riyadh 11451, Saudi Arabia; 10 Department of Pharmacy Practice, College of Pharamcy, AlMaarefa University, Ad Diriyah 13713, Saudi Arabia; mghoneim@mcst.edu.sa (M.M.G.); mrowaili@mcst.edu.sa (M.A.); 11 Department of Clinical Laboratory Science, Faculty of Applied Medical Science, Najran University, Najran 61441, Saudi Arabia; a-21-ksa@hotmail.com (A.A.A.A.); mmalshahrani@nu.edu.sa (M.M.A.); 12 Department of Botany, Soban Singh Jeena University, Almora 263601, Uttarakhand, India

**Keywords:** Eg5, lung cancer, secondary metabolites, fungi, machine learning, molecular docking, Indian Himalayan Region

## Abstract

Among the various types of cancer, lung cancer is the second most-diagnosed cancer worldwide. The kinesin spindle protein, Eg5, is a vital protein behind bipolar mitotic spindle establishment and maintenance during mitosis. Eg5 has been reported to contribute to cancer cell migration and angiogenesis impairment and has no role in resting, non-dividing cells. Thus, it could be considered as a vital target against several cancers, such as renal cancer, lung cancer, urothelial carcinoma, prostate cancer, squamous cell carcinoma, etc. In recent years, fungal secondary metabolites from the Indian Himalayan Region (IHR) have been identified as an important lead source in the drug development pipeline. Therefore, the present study aims to identify potential mycotic secondary metabolites against the Eg5 protein by applying integrated machine learning, chemoinformatics based in silico-screening methods and molecular dynamic simulation targeting lung cancer. Initially, a library of 1830 mycotic secondary metabolites was screened by a predictive machine-learning model developed based on the random forest algorithm with high sensitivity (1) and an ROC area of 0.99. Further, 319 out of 1830 compounds screened with active potential by the model were evaluated for their drug-likeness properties by applying four filters simultaneously, viz., Lipinski’s rule, CMC-50 like rule, Veber rule, and Ghose filter. A total of 13 compounds passed from all the above filters were considered for molecular docking, functional group analysis, and cell line cytotoxicity prediction. Finally, four hit mycotic secondary metabolites found in fungi from the IHR were screened viz., (−)-Cochlactone-A, Phelligridin C, Sterenin E, and Cyathusal A. All compounds have efficient binding potential with Eg5, containing functional groups like aromatic rings, rings, carboxylic acid esters, and carbonyl and with cell line cytotoxicity against lung cancer cell lines, namely, MCF-7, NCI-H226, NCI-H522, A549, and NCI H187. Further, the molecular dynamics simulation study confirms the docked complex rigidity and stability by exploring root mean square deviations, root mean square fluctuations, and radius of gyration analysis from 100 ns simulation trajectories. The screened compounds could be used further to develop effective drugs against lung and other types of cancer.

## 1. Introduction

Cancer is the second-leading cause of death globally after cardiovascular disease, and it is accountable for a 19.3 million increase in cases with 10 million deaths in 2020. Worldwide, the most commonly diagnosed cancer is female breast (11.7%), followed by lung cancer (~11.4%), colorectal cancers (10.0%), liver cancer (8.3%), stomach cancer (7.7%), and prostate cancer (7.3%) [1]. Being the second-leading cause of cancer-related deaths in developed countries, lung cancer is mounting at alarming rates in developing countries and low-middle-income countries, responsible for ~1.3 million deaths per year [2]. The over-expression of Eg5 has been reported in lung cancer and has an essential role in cell migration and angiogenesis impairment [3,4,5,6]. In this context, the mitotic kinesin, Eg5, is a promising target as it has a critical role in assembling the mitotic spindle during cell division. Kinesin superfamily proteins or KIF11/Eg5 are microtubule-based motor proteins that generate directional movement along microtubules. KIFs are core proteins, and these are not only essential for intracellular transport but are also important for various cellular and morphology functions [7]. KIF11/Eg5 is also identified as a prognostic factor. Overall, it indicates poor survival and the worst progression-free survival in lung cancer patients. Its knockdown is reported for induced G2/M phase arrest and improved apoptosis in lung cancer cells [8]. Various studies reported that the natural compounds inhibit the abnormal Eg5 effect and minimize tumors in patients suffering from lung cancer [9,10]. Therefore, Eg5 is a good target for anti-cancer drug development, and natural products may provide a huge source of compounds with therapeutic potential. With the above context, several fungal-derived natural products, such as lovastatin, echinocandin B, and cyclosporine A, are well reported with excellent therapeutic potential. Currently, several fungal secondary metabolites from the Indian Himalayan Region (IHR) have been identified as a vital source of potent therapeutic compounds. A diverse structural class of secondary metabolites, including aromatic compounds, amino acids, anthacenones, butanolides, butenolides, cytochalasans, macrolides, naphthalenones, pyrones, and terpenes, are produced by fungi [11]. Therefore, in the present study, mycotic secondary metabolites are screened for their anti-lung cancer property targeting the Eg5 protein. Several in silico techniques were used for screening, based on machine learning, chemo-informatics, and molecular dynamics simulation.

Currently, machine learning (ML) techniques are widely used in the cancer drug discovery process, including structure-based molecular docking, deep learning, and ligand-based cheminformatics modeling. ML also applies to proteo-chemometrics modeling, cell phenotype data, transcriptomics, and electronic health records (EHRs) based ML [12]. Drug development using artificial intelligence (Al), such as ML, is less costly and more time effective than the rational drug discovery process. In the drug discovery market, Al is projected to reach USD 1434 million by 2024 from USD 259 million in 2019 at a compound annual growth rate (CAGR) of 40.8% [13]. The machine learning-based quantitative structure-activity relationships (QSAR) models are widely used nowadays for screening huge compounds to predict potential inhibitors against diverse diseases. These types of models codify the chemical 3D structure based on molecular descriptors, such as constitutional, functional groups, geometrical, thermodynamic, topological, and quantum mechanical. However, the development of a QSAR model with high accuracy is a challenging task that depends on the correct analysis and selection of computed descriptors, application of proper classifier, and proper statistical understating of the model [14]. In the present work, machine-learning-based QSAR modeling, molecular docking, and other chemoinformatics-based screening techniques and molecular dynamics simulation were employed for screening natural inhibitors against the Eg5 protein targeting lung cancer.

## 2. Methods

### 2.1. Machine Learning Model Development and Screening

In the present work, the complete PubChem Human A549 Lung Tumor Cell Growth Inhibition Assay (AID: 317) data set of percentage inhibition for the cancer line was considered for model development [15]. The assay consisted of 3317 compounds in total, out of which for model development, 278 compounds (50% growth inhibition at 10 uM) were considered as active, and 294 compounds with negative or not having growth inhibition capacity were considered as inactive (Supplementary Materials, Table S1).

For screening, a structural library of 1830 mycotic secondary metabolites from 184 medicinal fungi was retrieved from the Medicinal Fungi Secondary Metabolite And Therapeutics (MeFSAT) database [16]. All compounds within the model and screening data set were further converted from the three-dimensional (3D) standard data format (SDF) to the molecular-input line-entry system (SMILES) format with the help of the O’babel software [17]. Thereafter, molecular descriptors were calculated for the compounds. Molecular descriptors generally encode various information about molecular fragmentation. A total of 2633 molecular descriptors were obtained, including one-dimensional (1D), two-dimensional (2D), and three-dimensional (3D) fingerprint features using the PaDEL-descriptor software [18]. Thereafter, both datasets, i.e., the CHEMBL dataset (AID 371) and the MeFSAT dataset were subjected to the Waikato Environment for Knowledge Analysis (WEKA) software [19] for building the predictive machine-learning model and screening. The CfsSubsetEval module implemented in WEKA was used for the significant descriptor selection. The descriptors with zero values were excluded followed by the removal of highly correlated descriptors. A total 16 descriptors from 8 descriptor types selected by the CFS Subset Evaluator were considered for the model development. Selected descriptor types include autocorrelation (ATSC6i, AATSC8s, GATS7c, GATS1e, GATS1p, GATS2i), basic group count (nBase), burden-modified eigenvalues (SpMin3_Bhe, SpMin1_Bhs), crippenLogP, atom type electrotopological state (nHsOH, SssNH, maxHCsatu), information content (BIC2), ring count (nFRing), and XLogP. Among them are the autocorrelation descriptors pool chemical information given by property values in specified molecule regions and structural information. They are based on a conceptual segmentation of the molecular structure and the application of an autocorrelation function to molecular properties of different molecular regions [20]. The second descriptor class is nBase, which counts the number of basic groups in the compounds that tend to be protonated in the gastrointestinal tract. Basic compounds have higher polarity and low lipophilicity that limits passive absorption across bio-membranes [21]. Further, the third descriptor class, Burden modified eigenvalues, derived from the eigenvalues of a modified adjacency matrix, is chemically intuitive in that its elements relate to atomic and bonding properties of the compounds [22]. The fourth, CrippenLogP descriptor class, simply determines the log P of the compounds by fitting an extensive training set of 9920 molecules with r^2^ of 0.918 and σ of 0.677 [23]. The fifth descriptor class, atom type electrotopological state descriptors, characterized by atoms in molecules, is introduced as the electrotopological state index, which combines both the electronic character and the topological environment of each skeletal atom in a molecule. They help to identify atoms and regions in the molecule which are important for activity [24]. The BIC2 descriptors are vital for the count of bond information content index (neighborhood symmetry of 2-order). The second to last descriptor, nFRing, gives information about the number of fused rings in a compound. The last descriptor class, XLogP, calculates partition coefficients of solutes in octanol/water responsible for lipophilicity of compounds, a vital parameter responsible for its ability to cross the cell membrane [25].

To develop a machine-learning model the PubChem dataset was divided into 70:30 as a training and test set. The training set was made up of 374 compounds while the test set had 161 compounds (Supplementary Materials, Table S1). Five machine-learning algorithms, namely, random forest, J48, decision stump, random tree, and REPTree classifier with bagging (Bootstrap Aggregation) ensemble algorithm were applied individually to get the best screening model. Random forest is one of the most frequently used ensemble-learning algorithms in the field of in silico drug discovery. It generates multiple decision trees and for one test instance, and the classification of each tree is regarded as one vote, and the test descriptors were confirmed by integrating the prediction results of each tree (sub-classifier) [26]. In J48, the main root of the decision tree is established based on the gain and gain ratio values of the descriptors. In the decision stump algorithm, the classification process is performed by considering only one key feature in the sample set and has root node and leaves. In a random tree algorithm, trees are constructed using randomly selected samples from the descriptor data set and a random one is selected. Whereas, the REPTree creates rapid multiple decision trees, using information gain, prunes them using reduced error pruning and selects the best tree among all generated trees [27]. The accuracy of the models was evaluated by using the following statistical parameters [28]:

$$\mathrm{Binary}\;\mathrm{classification}\;\mathrm{accuracy}=\frac{\mathrm{TN}+\mathrm{TP}}{\left(\mathrm{TN}+\mathrm{TP}+\mathrm{FN}+\mathrm{FP}\right)},\;\;\mathrm{Sensitivity}=\frac{\mathrm{TP}}{\left(\mathrm{FN}+\mathrm{TP}\right)},\;\;\mathrm{Specificity}=\frac{\left(\mathrm{TN}\right)}{\left(\mathrm{FP}+\mathrm{TN}\right)},$$

Accuracy_random=TP+FN×TP+FP+TN+FN×TN+FPN^2

$$\mathrm{Matthews}\;\mathrm{correlation}\;\mathrm{coefficient}\;\left(\mathrm{MCC}\right)=\frac{\left(\mathrm{TN}\times \mathrm{TP}\right)-\left(\mathrm{FN}\times \mathrm{FP}\right)}{\sqrt[{}]{\left(\mathrm{FP}+\mathrm{TP}\right)\left(\mathrm{FN}+\mathrm{TP}\right)\left(\mathrm{FP}+\mathrm{TN}\right)\left(\mathrm{FN}+\mathrm{TN}\right)}},\;$$

TP = true positives; FP = false positives; TN = true negatives; FN = false negatives.

The best machine model was used as a predictive model to screen anti-lung cancer compounds from the library of 1830 mycotic secondary metabolites.

### 2.2. Drug-Likeness Screening and Molecular Docking

The DruLiTo-screening tool was further used to screen the ligands with drug-likeness properties [29]. Chemical descriptors such as atom molar refractivity (AMR), AlogP, logP, H-bond acceptor (HBA), H-bond donor (HBD), molecular weight (MW), number of rotatable bonds (nRB), number of atoms, number of acidic groups, rotatable bond count (RC), number of rigid bonds (nRigidB), nAtomRing, nHB, and total polar surface area (TPSA) for all of the ligands were calculated using the chemistry development kit (CDK) [30].

DruLiTo screened the ligands based on the various drug-likeness rules, such as Lipinski’s rule, CMC-50-like rule, Veber rule, and Ghose filter [31]. After screening through the machine-learning model, screened compounds were subjected to target-specific virtual screening. For that, the three-dimensional structure of the target protein, Eg5 (PDB id: 4BXN), was downloaded from an online protein data bank portal [32]. The target protein and machine-learning screened compounds were docked using the PyRx graphic user interface (GUI), which utilizes the AutoDockVina virtual screening program [31]. All screened ligands were docked in the active site of the Eg5 protein with coordinates of X = 11.82, Y = 37.46, and Z = −9.79 to obtain protein–ligand complexes. The compound, N-(3-aminopropyl)-N-[(1R)-1-(3-benzyl-7-chloro-4-oxo-4H-chromen-2-yl)-2-methylpropyl]-4, methylbenzamide, was considered as a reference compound for examining the necessary binding score. Further, the docked protein and ligand complexes were visualized using UCSF chimera [33]. The in-depth interactions were investigated using the protein–ligand interaction profiler server (PLIP) [34].

### 2.3. Functional Group Analysis and Cell Line Cytotoxicity Prediction

The functional group analysis method was used to functionalize the physical and chemical properties of small groups and atoms that exhibit a distinctive chemical nature when in their original form. The functional group’s frequencies of the screened compounds were analyzed in ‘R’ (version 3.4.3) [35] using the ChemMineR [36] package followed by standard deviation analysis. Five established inhibitors against Eg5 (CID: 132472242, 53494981, 11609157, 11553595, and 11521919) were also subjected to function group analysis for comparison. Functional groups, such as aromatic rings, rings, carboxylic acid esters, and carbonyl, were evaluated for both screened inhibitors and reference inhibitor groups [37]. Thereafter, virtually screened compounds from machine learning, docking, and functional group analysis were additionally executed to evaluate their cell line cytotoxicity prediction. This analysis was carried out by using CLC-Pred web facilities (CLC-Pred: in silico prediction of cytotoxicity for tumor and non-tumor cell lines (way2drug.com, accessed on 8 June 2021)). This estimation of web services was conducted based on the QSAR model build on the prediction of activity spectra for substances (PASS) tools (http://www.way2drug.com/PASSonline, accessed on 8 June 2021) and the training dataset produced based on ChEMBLdb cytotoxicity data [38,39].

### 2.4. Molecular Dynamics Simulation

The docked complexes of the final screened compounds were further checked for their binding stability to MD simulations using GROMACS 5.0 [40]. The CGenFF server was used to generate topologies and coordinate files. The CHARMM 36 force field was used for topologies of the complex structures of the ligand molecules [41]. The SPC/E water mode l40 was used for solvation of the complexes followed by the addition of counterions for neutralization. The simulation was carried out up to 100 ns time in NVT (constant volume), as well as NPT (constant pressure), maintaining the temperature at 300 K and pressure 1 bar. Finally, the stability of the protein–ligand complexes was analyzed by considering parameters, such as RMSD (root mean square deviations) and radius of gyration (Rg).

## 3. Results

### 3.1. Performance Comparison of Different ML Classifiers and Data Set Screening

**Figure 1:**
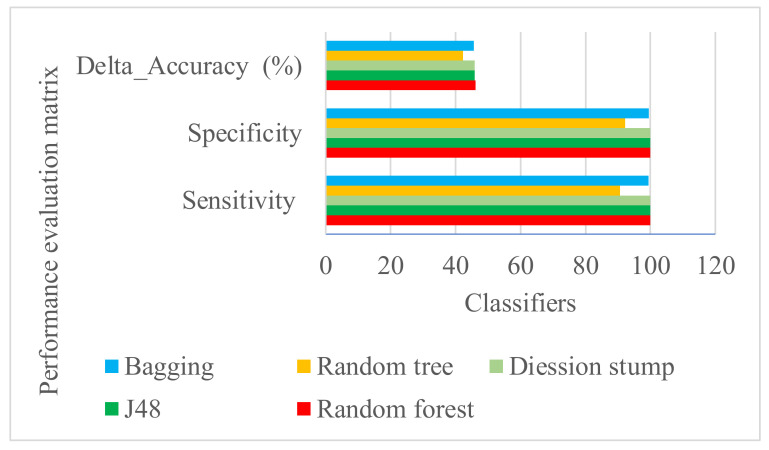
Statistical performance of different classifiers used for the development of screening model in the training set.

The performance of the ML algorithms varies with the type of training data set of the model. Therefore, for identification of the best-performing algorithm on the lung cancer inhibitor data set, five classifiers were evaluated for their classification accuracy, namely, random forest, J48, decision stump, random tree, and REP tree classifier with bagging. The performance of the models was assessed with the evaluation of various statistical parameters, viz., correctly classified instances, incorrectly classified instances, Kappa statistic, mean absolute error, root mean square error, Matthews correlation coefficient (MCC), and receiver operating characteristic curve (ROC) area (Figure 1, Table 1). Among all the classifiers, the random forest classifier with the highest sensitivity (1), Kappa statistics of 0.94, and ROC of 0.99 area in the training set was selected for the final screening model (Supplementary Materials, Table S2). The model also performed best in the test set (Supplementary Materials, Table S3). It was able to classify 97.05% of instances of the supplied training set data correctly with a mean out-of-bag (OOB) error of 0.07 (MAE). In the bagging (REPTree) model out-of-bag estimates, 96.52% instances were correctly classified with 0.18 root mean squared error (RMSE). However, bagging (REPTree) and decision stump showed lower sensitivity than random forest. The random tree classifier showed the lowest performance with sensitivity of 0.90, specificity of 0.92, and accuracy of 0.09 (K = 0–4, randomly chosen attributes at each node). The random forest classifier consists of a collection of tree-structured classifiers {h(x, Θ k), k = 1…}, where each tree creates a unit vote against the most popular class at input xand the {Θ k} are independent identically distributed random vectors [42]. The model of the current work was obtained based on the default mtry (number of randomly drawn candidate variables) for classification, square root of the number of descriptors, *p* = 16, and 100 trees in the forest (ntree). The “forest” built by the model, is an ensemble of decision trees, usually trained with the “bagging” method. The bagging algorithm simulates a defined method before applying a certain training set to remove the instability of learning techniques. The initial training set data is improved by removing some samples and duplicating others in its place of sampling a new training dataset each time by random sampling. The process of sampling necessarily rejects some of the instances and duplicates the others. Bagging simply resamples the native training data instead of creating autonomous data sets from the domain. It makes a combined model that mostly performs better than the single model made from the native training data [43]. The selected model classifies 319 mycotic secondary metabolites with active potential out of 1830 compounds.

### 3.2. Drug Likeness and Molecular Docking

**Figure 2:**
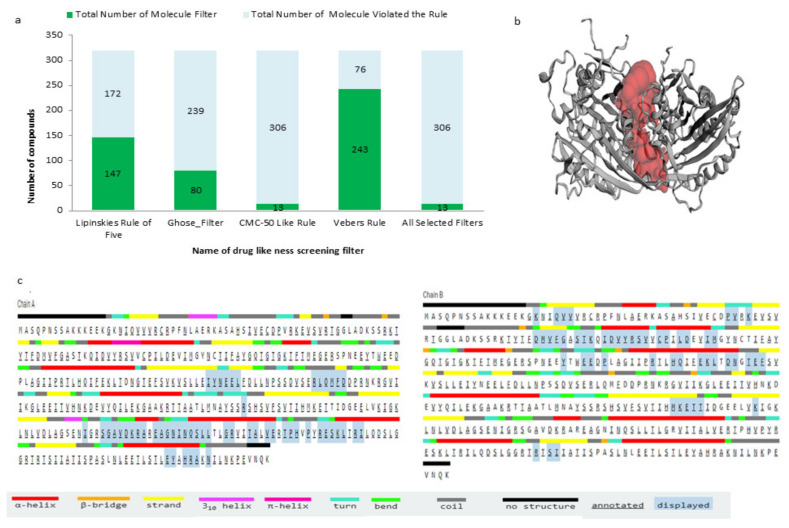
(**a**) Drug-likeness filters used for screening compounds. (**b**,**c**) Active site prediction of Eg5 protein for molecular docking.

Screened compounds from ML were further checked for their drug-likeness property by applying four pharmacological filters, namely, Lipinski’s rule, CMC-50-like rule, Veber rule, and Ghose filter. According to Lipinski’s rule, a drug-like compound should have LogP ≤ 5, molecular weight ≤ 500, number of hydrogen bond acceptors ≤ 10, and number of hydrogen bond donors ≤ 5. Whereas, according to the CMC-50-like rule, a drug-like compound must have logP (ALOGP) between 1.3 and 4.1, molar refractivity (AMR) between 70 and 110, molecular weight (MW) between 230 and 390, and the number of atoms (nAT) between 30 and 55. To qualify the Veber rule and Ghose filter, compounds should have rotatable bonds < 12 and polar surface area < 140, logP in the −0.4 to +5.6 range, molar refractivity from 40 to 130, molecular weight from 180 to 480, and the number of atoms from 20 to 70, respectively. A total of 13 compounds out of 319 (Supplementary Materials, Table S4) compounds were able to ensure all the above filters and were considered for molecular docking to check their binding potential with the Eg5 protein (Table 2 and Figure 2). Of those 12, compounds having the lowest binding energy (highest inhibitory potency) were screened further based on the binding energy of the reference compound.

### 3.3. Functional Group Cell Line Cytotoxicity and Binding Pattern of Screened Molecules

**Figure 3:**
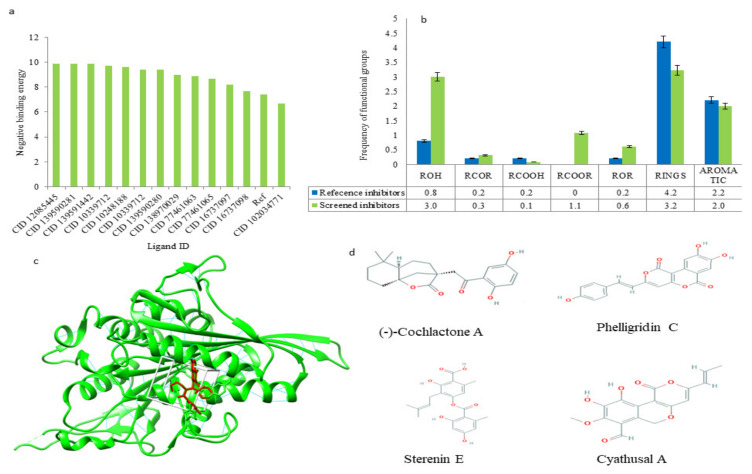
(**a**) Binding free energy of the screened ligands through molecular docking. (**b**) Functional group frequency comparison between established inhibitors and screened compounds. (**c**) Binding insights of the screened ligand, Phelligridin-C, with Eg5 protein. (**d**) 2D structure of screened compound.

**Figure 4:**
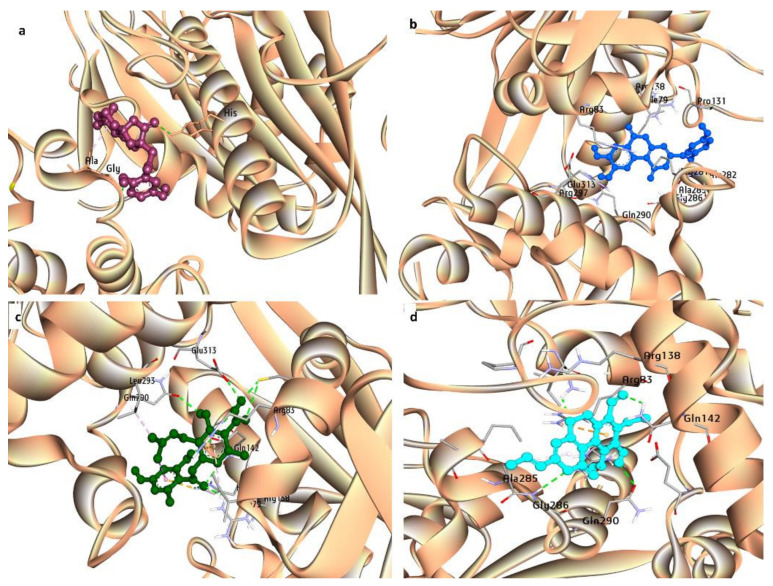
Interaction profile of screened ligands with Eg5 protein: (**a**) (-)-Cochlactone A, (**b**) Phelligridin-C, (**c**) Sterenin E, and (**d**) Cyathusal A.

During the analysis of the functional group frequency among some of the established inhibitors of Eg5, viz., the presence of aromatic rings, rings, carboxylic acid esters, and carbonyl groups were shown. A similar frequency pattern was observed in the screened compounds (Figure 3), which reflects their excellent Eg5 inhibitory property. The high frequency of alcohol (ROH), ketone (RCOR), esters (RCOOR), and ether (ROR) groups were analyzed in screened inhibitors as compared to reference inhibitors (Figure 3). The groups, such as alcohol (ROH), ketone (RCOR), and esters (RCOOR), indicate more H-bonding capacity of the compounds or more water solubility. Simultaneously, screened compounds also exhibited a high presence of rings and aromatic rings that prove their strong drug-likeness potential. Aromatic rings commonly promote Van der Waals interactions with the binding site atoms of the protein [44]. Further, the inhibition potential of the compounds in cell lines was checked, and four compounds, namely, (-)-Cochlactone A, Phelligridin C, Sterenin E, and Cyathusal A, were predicted to be active against the lung cancer cell lines, NCI-H226, NCI-H522, A549, and NCI-H187 (Supplementary Materials, Table S5), respectively. The binding profiles of the screened compounds were also checked and all of the above compounds were found with efficient binding potential and can form diverse bonds, including hydrophobic interactions, H-bonds, salt bridges, and π–cation interactions (Table 3, Figure 4). Hydrophobic interactions are vital as they have an energetic contribution to the stability of the protein–ligand complex [45], whereas H-bonds are necessary for enzymatic catalysis to stabilize ligands in the binding pocket [46]. All the screened compounds were found to be able to bond within the switch II (Leu266–Asn289) and α-helix 4 (Gln290–Val303) regions of the allosteric site of the Eg5 protein. ATP-competitive inhibitors of Eg5, including the biphenyl derivatives, have been reported to bind with the protein between the α4 and α6 helices. In Eg5, switch I forms repeated contacts with the γ-phosphate of ATP while switch II offered a β-hairpin conformation [47].

### 3.4. Binding Stability Analysis of the Screened Compounds

**Figure 5:**
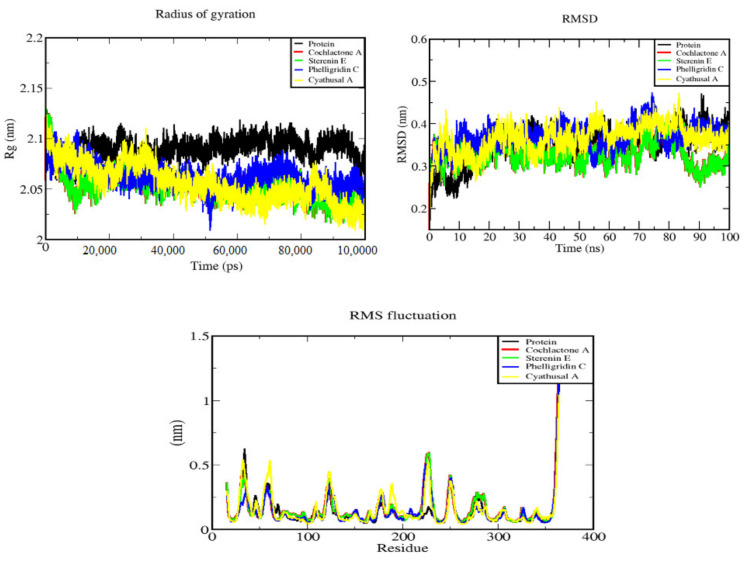
Curves illustrating the behavior of the interactions of screened compounds with EG5 protein in the form of Rg, RMSF, and RMSD during MD simulation.

Finally, the structural stability of the screened ligands with the protein was studied by molecular dynamics (MD) simulations. MD simulation was used to predict the movement of every atom in a protein and protein ligand complex over time, based on the general physics model of the governing interatomic interactions. The structural compactness of the screened compounds was assessed by calculating the radius of gyration (Rg) defined as the distribution of atoms of a protein around its axis. All the systems, such as protein and the four complexes, showed stability in the MD simulation approach towards 100 ns time scale between 1.7 and 1.8 nm, reflecting the high structural compactness of the protein, as well as the inhibitor. The lower degree of fluctuation with its consistency throughout the simulation designates more compactness and rigidity of the protein ligand complex. The Rg of Cochlactone A is found to be almost stable in terms of consistency of fluctuations throughout the simulation. Deviations of the conformational stability of macromolecules from the backbone structure to the initial structure were evaluated to check their stability by calculating the root mean square deviation (RMSD). All complexes showed RMSD values between 0.31 and 0.37 nm, and the value starts to increase after 10 ns. The compound, Sterenin E, showed a decreasing pattern after 80 ns. Further, the flexibility of proteins along with all the complexes throughout the simulation process was evaluated by calculating the root mean square fluctuations (RMSF). It indicates the occurrence of local changes along with the protein structure at the selected temperature and pressure. The Cyathusal A_EG5 complex showed the highest RMSF (0.14 nm) values followed by other screened compounds. It can also be understood from Figure 5 that the binding of Cyathusal A makes the protein most flexible in all areas in contrast to the other complexes (Supplementary Materials Table S6, Figure 5).

## 4. Discussion

The IHR is well known in the field of drug discovery as a treasure house for numerous natural compounds with extensive therapeutic potential. However, the endophytic microbial biodiversity of the area is less explored to date. The endophytic fungus isolated from the medicinal plants of IHR contain several anti-cancer compounds, e.g., Taxol found in Annulohypoxylon sp. Isolated from the Himalayan yew (*Taxus wallichiana* Zucc.), Vinblastine produced by *Curvularia verruculosa,* and Vincristine by *Fusarium oxysporum* isolated from *Catharanthus roseus*, *Podophyllotoxin* from *Phialocephala fortinii*, Camptothecin from *Entrophospora infrequens* from the plant *Nothapodytes nimmoniana* etc. [48,49].

Based on the present work, four mycotic secondary metabolites were screened, namely, (−)-Cochlactone A, Phelligridin C, Sterenin E, and Cyathusal A. (−)-Cochlactone A is an alkyl-phenyl ketone, an aromatic compound containing a ketone replaced by one alkyl group and a phenyl group. In 2018, it was found in the fungus *Ganoderma cochlear*, an edible and medicinal fungus. The compound has a bicycle [4.4.0] decane ring system with a γ-lactone fragment and possesses anti-inflammatory activity [50]. Other species, such as *Ganoderma lucidum* from the same genus, is well known as a medicinal fungus from the IHR [51]. The second screened compound, Phelligridin C, is a furo [3, 2-c] pyran-4-one derivative reported from the fungus *Phellinus igniarius* [52]. It is recorded to have cytotoxicity against a human lung cancer cell line. The genus is also reported from the IHR of the Himachal Pradesh and Uttarakhand states of India [53]. The third screened compound, Sterenin E, is a polyketide that has α-glucosidase inhibitory activity from the mushroom, *Stereum hirsutum* [54]. Naturally, the fungi are known from the Hmuifang forest and Tanhril forest of the IHR from Mizoram, Northeast India [55]. The fourth screened compound, Cyathusal A, is recorded from the mushroom, *Cyathus stercoreus,* and has free-radical-scavenging activities on the 2,2-diphenyl-1-picrylhydrazyl (DPPH) radical and is thus a potent antioxidant [56]. The fungus is naturally found in the humicolous soil in the Khasi hills of Meghalaya, the upper Shillong area of the IHR [57].

## 5. Conclusions

The Kinesin protein, Eg5, is a well-established anti-lung cancer target for its role in the assembling of the mitotic spindle directional movement during cell division. Therefore, in the current work, four natural anti-lung cancer inhibitors, viz., (−)-Cochlactone A, Phelligridin C, Sterenin E, and Cyathusal A, have been identified against lung cancer targeting the Eg5 protein by applying the machine-learning and chemoinformatics approaches. All the screened compounds were of mycotic origin and found in the fungi from the IHR. Being of a natural origin, the screened mycotic secondary metabolites may have fewer side effects than other chemotherapeutic drugs used against lung cancer and can be obtained easily by culturing the fungi in the laboratory. They are potential anti-inhibitors as screened by the machine-learning model designed using a lung cancer inhibitor bioassay and have efficient binding potential with the Eg5 protein. Additionally, they have passed all the selected drug-likeness filters and have an excellent presentation of diverse functional groups with pharmacological importance and a stable-binding profile during the MD simulation process, which proves their potential as an anti-cancer drug candidate.

## Figures and Tables

**Table 1:** Comparison of performance of different classifiers for development of screening model in the training set.

Classifier Name	Correctly Classified Instances % (Value)	Kappa Statistic	Mean Absolute Error	Root Mean Square Error	MCC	ROC Area
Random forest	97.0588	0.9401	0.08	0.1731	0.942	0.989
J48	96.7914	0.9346	0.05	0.175	0.937	0.964
Decision stump	96.7914	0.9346	0.06	0.175	0.937	0.947
Random tree	92.7807	0.8544	0.07	0.2687	0.855	0.928
Bagging (REP tree)	96.5241	0.9292	0.06	0.1844	0.931	0.96

**Table 2:** Pharmacological indices of the screened ligands by Lipinski’s rule, CMC-50 like rule, Veber rule, and Ghose filter.

Title *	1	2	3	4	5	6	7	8	9	10	11	12	13
Pharmacological Indices													
MW	358	358	372	372	330	346	378	378	380	364	386	370	358
logp	4	4	2	2	2	2	1	1	2	2	4	4	2
Alogp	1	1	1	1	0	−1	1	1	0	1	3	3	0
HBA	5	5	6	6	7	8	6	6	8	7	7	6	7
HBD	2	2	3	3	2	3	4	4	4	3	4	3	3
TPSA	84	84	96	96	102	123	107	107	134	113	124	104	113
AMR	98	98	105	105	90	91	113	113	106	104	109	108	87
nRB	3	3	4	4	3	3	3	3	2	2	6	6	5
nAtom	52	52	51	51	38	39	46	46	40	39	50	49	55
nAcidicGroup	0	0	0	0	0	0	0	0	0	0	1	0	0
RC	4	4	3	3	3	3	4	4	4	4	2	2	2
nRigidB	26	26	25	25	23	24	28	28	29	28	23	22	21
nAromRing	1	1	1	1	1	1	3	3	2	2	2	2	0
nHB	7	7	9	9	9	11	10	10	12	10	11	9	10
SAlerts	4	4	5	5	5	5	0	0	4	4	3	4	2

* Compound CID: 1-138970029, 2-139591442, 3-139590281, 4-139590280, 5-16737098, 6-16737097, 7-12085445, 8-54586497, 9-10339712, 10-10248188, 11-77461063, 12-77461065, and 13-10203477.

**Table 3:** Interaction profile of screened ligands with Eg5 protein.

Ligand Name	Hydrophobic Interactions			Hydrogen Bond		Other			
	Residue	AA	Distance	Residue	AA	Distance	Residue	AA	Distance
(−)-Cochlactone-A	79B	ILE	3.85	286A	GLY	1.85	Salt Bridges		
	-	-	-	286A	GLY	2.16	138B	ARG	4.45
	131B	PRO	3.53	297A	ARG	3.13	141B	HIS	5.14
	-	-	-	-	-	-	-	-	-
	285A	ALA	3.42	-	-	-	-	-	-
	-	-	-	-	-	-	-	-	-
Phelligridin-C	79B	ILE	3.92	83B	ARG	3.27	π–Cation Interactions		
	125B	TYR	3.72	142B	GLN	2.55	83B	ARG	4.97
	-	-	-	286A	GLY	1.92			
	131B	PRO	3.83	290A	GLN	2.5	Salt Bridges		
	285A	ALA	3.29	297A	ARG	2.2	138B	ARG	4.1
Sterenin-E	79B	ILE	3.43	83B	ARG	3.09	π–Cation Interactions		
	82B	TYR	3.52	141B	HIS	2.69	83B	ARG	5.19
	-	-	-	142B	GLN	2.72	Salt Bridges		
	-	-	-	142B	GLN	2.66	138B	ARG	4.74
	293A	LEU	3.38	287A	ASN	2.55	141B	HIS	5.08
	-	-	-	290A	GLN	2.66	-	-	-
	-	-	-	297A	ARG	3.36	-	-	-
Cyathusal-A	79B	ILE	3.73	83B	ARG	3.15	π–Cation Interactions		
	-	-	-	138B	ARG	2.37	83B	ARG	5.11
	285A	ALA	3.92	142B	GLN	2.51	-	-	-
	-	-	-	290A	GLN	2.5	-	-	-
	-	-	-	297A	ARG	2.45	-	-	-

## Data Availability

Not applicable.

## Supplementary Materials

Table S1: Percent growth inhibition data of active and inactive compounds used for ML, Table S2: Training set performance matrix, Table S3: Test set performance matrix, Table S4: Pharmacological properties of screened ligands by machine learning, Table S5: Percent growth inhibition data of active and inactive compounds used for ML, Table S6: Binding stability parameters of the screened compounds during molecular dynamics simulation.

## Sample Availability

Samples of the compounds are not available from the authors.

## Abbreviations

AL	Artificial Intelligence
AMR	Atom Molar Refractivity
CAGR	Compound Annual Growth Rate
CDK	Chemistry Development Kit
CSF	Correlation-Based Feature Selection
ER	Estrogen Receptor
GUI	Graphic User Interface
HER	Electronic Health Records
IHR	Indian Himalayan Region
KIF-11	Kinesin Family Member-11
MCF-7	Michigan Cancer Foundation-7
MD	Molecular Dynamics
MeFSAT	Medicinal Fungi Secondary Metabolite And Therapeutics
ML	Machine Learning
NIC	National Cancer Institute
PDB	Protein Data Bank
PLIP	Protein Legend Interaction Profiler
QED	Quantitative Estimate of Drug-Likeness
QSAR	Quantitative Structure-Activity Relationship
RG	Radius of Gyration
RMSD	Root Mean Square Deviations
ROC	Receiver Operating Characteristic Curve
SMILES	Standard Data Format (SDF) To Molecular-Input Line-Entry System
TPSA	Total Polar Surface Area
USD	United States Dollar
WEKA	Waikato Environment for Knowledge Analysis
